# The Potential of Calorie Restriction and Calorie Restriction Mimetics in Delaying Aging: Focus on Experimental Models

**DOI:** 10.3390/nu13072346

**Published:** 2021-07-09

**Authors:** Emiliana Giacomello, Luana Toniolo

**Affiliations:** 1 Department of Medicine, Surgery and Health Sciences, University of Trieste, 34149 Trieste, Italy; 2 Laboratory of Muscle Biophysics, Department of Biomedical Sciences, University of Padova, 35131 Padova, Italy

**Keywords:** aging, life-span, health-span, calorie restriction, calorie restriction mimetic, resveratrol, experimental models

## Abstract

Aging is a biological process determined by multiple cellular mechanisms, such as genomic instability, telomere attrition, epigenetic alterations, loss of proteostasis, deregulated nutrient sensing, mitochondrial dysfunction, cellular senescence, stem cell exhaustion, and altered intercellular communication, that ultimately concur in the functional decline of the individual. The evidence that the old population is steadily increasing and will triplicate in the next 50 years, together with the fact the elderlies are more prone to develop pathologies such as cancer, diabetes, and degenerative disorders, stimulates an important effort in finding specific countermeasures. Calorie restriction (CR) has been demonstrated to modulate nutrient sensing mechanisms, inducing a better metabolic profile, enhanced stress resistance, reduced oxidative stress, and improved inflammatory response. Therefore, CR and CR-mimetics have been suggested as powerful means to slow aging and extend healthy life-span in experimental models and humans. Taking into consideration the difficulties and ethical issues in performing aging research and testing anti-aging interventions in humans, researchers initially need to work with experimental models. The present review reports the major experimental models utilized in the study of CR and CR-mimetics, highlighting their application in the laboratory routine, and their translation to human research.

## 1. Introduction

The rapid growth of the world’s aging population (https://population.un.org/wpp/; reporting the World population prospect of 2019, accessed on March 2021) has motivated a large effort in the investigation of the mechanisms underlying aging, and in the search of possible countermeasures.

Aging is characterized by two connected aspects: the malfunctioning of multiple basic biological processes and the parallel functional decline of the individual. Actually, the alteration of the molecular mechanisms regulating basic processes can increase the risk of developing chronic diseases (e.g., cardiovascular disease, diabetes, cancer, and neurodegeneration), meanwhile the functional decline of the individual contributes to a negative outcome to health-challenging situations.

More recently, it has been suggested that aging is determined by nine biological processes, which are: genomic instability, telomere attrition, epigenetic alterations, loss of proteostasis, deregulated nutrient sensing, mitochondrial dysfunction, cellular senescence, stem cell exhaustion, and altered intercellular communication [1]. Each distinct hallmark of aging has been identified based on the following three characteristics: (1) it is displayed during normal aging; (2) its experimental intensification accelerates aging; and (3) its experimental abatement delays aging [1]. The accumulation of the effects of this damage over time inevitably leads to cell death.

Among the multiple alterations that have a profound impact on aging, the nutrient sensing cell pathways have recently captured much interest thanks to their potential as therapeutic targets in the prevention of age-related diseases, and the extension of the healthy life-span. The nutrient sensing pathways are mainly regrouped in the IGF (insulin-like growth factor)/insulin, the TOR (target of rapamycin), and the AMPK (AMP-Activated Protein Kinase) pathways [2]. It has been extensively shown that, the presence of cellular nutrients induces the stimulation of insulin receptor and IGF-1R generating a phosphorylation cascade that activates AKT (Ak transforming), which induces glucose metabolism through GSK-3β, suppresses a wide range of cellular responses via the FOXO (Forkhead box) transcription factor, and stimulates protein synthesis by activating TORC1 (TOR Complex1), leading to protein synthesis and cell growth. Moreover, IGF-1R dependent signal, activates the Ras/MAPK signaling pathway, which results in cell proliferation. Contrarily, when the cell is deprived of nutrients or in low energetic conditions, AMPK levels increase and inhibit protein synthesis through TORC1, reducing anabolic processes and inducing mitochondrial respiration [2].

Accordingly, data from different experimental models have largely demonstrated that the mutations that induce life-span extension are associated with an altered activity of the above-listed signaling pathways [3].

Interestingly, the extension of the life-span upon inhibition of the nutrient sensing signaling pathways, has also been associated to the physiological condition induced by calorie restriction (CR). Actually, CR, which consists of the reduction in the caloric intake without malnutrition, has been reported as a robust intervention to promote life-span elongation and healthy aging in rodents at the beginning of last century [4], and has been further suggested to have similar effects in humans [5,6]. CR regimens have been shown to induce metabolic adaptations, such as reduced oxidative stress and improved inflammatory response [7,8], that ultimately result in better life- and health-spans. Studies performed on experimental models allowed to attribute the life prolongation effects to the modulation of the IGF-1 [9,10], TOR [11], and AMPK signaling pathways [12], but also to other targets, such as the above mentioned FOXO that stimulates protein synthesis, NfkappaB, which is involved in the inflammatory response, and Nfr2 that is implicated in mitochondrial biogenesis [2,13,14,15]. Moreover, recent work brought to the identification of the Sir2/SIRT1 NAD-dependent histone deacetylase, which is involved in the chromatin silencing pathway, as the key regulator of life- and health-span extension induced by CR [16,17,18]. The identification of the regulatory properties of Sirtuins, together with the evidence that this pathway is conserved among different species [16], has provided to this molecular pathway a target role, pharmacologically adjustable, for the amelioration of the health-span, especially in those individuals who cannot afford CR interventions. As a consequence, at present there is a wide field of research focused on the investigation of natural and synthetic compounds (CR-mimetics), aiming at improving life- and health-span in humans [19,20,21]. Among the most known, the natural polyphenol resveratrol, a potent SIRT1 activator is largely investigated in both experimental models and humans [22,23,24,25].

The study of the aging mechanisms and the possible countermeasures to contrast them in humans is challenging due to the long duration of the aging process itself. Longitudinal studies are difficult to perform because they need an important effort, traceability and continuity that, trivially, are very problematic to maintain for a long time. On the other side, cross-sectional studies can be influenced by multiple factors, especially if we consider that in the last century several parameters characterizing the life quality, as the socio-sanitary conditions, the nutritional regimens and psychophysical activities, have undergone dramatic changes. In addition, the heterogeneity of population characteristics can further complicate the analyses.

Mainly for these reasons, but also for ethical issues, the study of aging is conducted on experimental models. Unfortunately, experimental models are not devoid of pitfalls, flaws or obstacles. Multiple factors must be considered when planning experiments to investigate the influence of CR and CR mimetics on the aging process. Although theoretical life-span curves are quite homogeneous in shape across several experimental models, they can be enormously different in length [26], and cannot provide information on the real health condition (health-span) of the experimental model [27]. Furthermore, aging can induce diverse modifications at the tissue or organ level depending on the experimental model used [28,29]. Not least, CR mechanisms underlying life-span extension can differ among species [30,31].

The present review aims at providing an overview of the major experimental models utilized in aging research, highlighting the characteristics that allow their use in the study of CR and CR mimetics, and their translation to human research, with a daily-laboratory routine perspective.

## 2. *Saccharomyces cerevisiae*

The first studies on aging in the budding yeast *Saccharomyces cerevisiae* date back to 60 years ago [32], since then its application in aging research has been continuously active. More recently, at the beginning of the 21st century, the use of yeast in aging research saw an important outburst thanks to the discovery of Sirtuins, a class of histone deacetylases involved in life-span regulation.

*Saccharomyces cerevisiae* is a unicellular eukaryotic organism with 6000 completely sequenced genes [33,34], and with a short life cycle. Yeast cells proliferate in both haploid or diploid state depending on nutrients abundance. With a sufficient nutritional supply, cells proliferate in a diploid state with a cell cycle of 2 h, while they enter meiosis and spore formation upon nutrient withdrawal. Spores can survive from hours to months, and in favorable conditions haploid a and alpha spores can proliferate and mate to form diploid cells. The simple laboratory equipment required, the short generation time, and a well-characterized genome displaying high similarities with mammalian cells, easily modifiable by means of genetic approaches, make *Saccharomyces cerevisiae* an optimal tool to dissect the molecular mechanisms of multiple pathways involved in aging.

The life cycle and senescence in the budding yeast has been defined exploiting two different experimental protocols, which measure two different biological properties. These are the replicative life-span and chronological life-span, respectively [32,35,36]. The first experimental protocol is based on the evidence that the budding capacity of a single mother-cell decreases with time. The second experimental protocol is based on the analysis of the growth to plateau concentration of a population in a liquid medium, and the estimation of percentage of viable or metabolic active cells [34].

Evidences showing that reduction in glucose availability in the growth medium from 2% to 0.01% induced an extension of *Saccharomyces cerevisiae* life-span, have made this organism an excellent model to study the fundamental mechanisms of life prolongation upon CR [17,37]. Interestingly, in 2000, studies from separate groups allowed the identification the Sir-2 NAD-dependent histone deacetylase pathway [17,18,38] as a key regulator of life-span extension. Only subsequently, the Sir2 pathway has been shown to be conserved among species [16].

At date, the signaling pathways regulating the replicative and the chronological life-span are better characterized. In fact, the RAS-PKA and Tor-Sch9 signaling pathways have been demonstrated to be consistent in both the replicative and the chronological life-span, while the effects of the modulation of Sir2 pathway on the chronological life-span are opposite to replicative life-span [17,39]. These evidences provide a further element to orient the researcher in the choice of the most appropriate experimental protocol.

Interestingly, the above-mentioned characteristics make the budding yeast very suitable for high-throughput methodologies, particularly in the screening of anti-aging compounds [40,41,42]. In this context, the work of Howitz and collaborators [20], reported the discovery of small molecules, such as resveratrol, able to activate Sirtuins and induce life-span extension, tracing the pace to the design and screening of new CR-mimetic compounds.

Although the yeast allows a precise characterization of some molecular pathways involved in aging, its application in the study of CR and CR-mimetics is limited, not only because it cannot provide information at the tissue and organ level, but also because CR mechanisms can differ among species [31]. Indeed, mammals contain seven homologs of the yeast Sir2, which have different cellular localization, different protein interactions and different biological function [30].

## 3. *Caenorhabditis elegans*

Since the 1970s the small nematode *Caenorhabditis elegans* is one of the most used organisms in aging research and in the study of genetics of aging [43,44]. Interestingly, it allowed the identification of the insulin/IGF-1 signaling pathway, and afterwards FOXO, as key regulators of the life-span extension [9,10,45]. Only subsequently, the insulin/IGF-1 pathway was found and better characterized in other model animals and humans [46,47,48].

In normal conditions (20 °C), *C. elegans* develops from an egg and undergoes four larva stages (L1–L4) to become a reproductive adult hermaphrodite worm in three days. The mean life-span of the *C. elegans* is around 15 days, and the maximum life-span around 27 days. In adverse conditions (i.e., temperature or nutrients restriction), worms after the larval stage L2 can enter an alternative developmental state named dauer larvae, which is stress and age-resistant [49,50]. When conditions become more favorable, dauer larvae can convert into a reproductive adult. The adult worm is a simple organism composed by about 1000 cells, that form distinct tissues and organs with a functional similarity to human organs [51,52]. With age progression, *C. elegans* worms reduce their activity, become less coordinate and can eventually stop moving in an age-dependent fashion [53]. Moreover, normal and transgenic *C. elegans* have shown to display some of the aging features as sarcopenia [54], and neurodegeneration [55,56].

From the laboratory routine perspective, *C. elegans* is an interesting experimental model since it is relatively easy to maintain, and the growth medium easily tunable [53]. The *C. elegans* genome is deciphered, easily modifiable, and with good association to human genes [57,58]. The presence of a RNAi library that covers about 80% of the genes allows extensive screens to detect genes involved in the modulation of life-span [59,60]. The life-spans of a population have generally no, or little, fluctuation, allowing the identification of factors that increase or reduce the life-span by 10–15% with statistical significance [60].

*C. elegans* has been adopted as the prominent model in aging research for several years because it has been found that mutations to genes that regulate the dauer stage correlate with life-span. For instance, the analyses of age-1 and daf-2 mutants, which displayed a longer life-span, led to the identification of the insulin/IGF-1 signaling pathway [9,10]. Subsequently, daf-16 mutants allowed the identification of the FOXO pathway, and revealed the importance of this pathway in improving the resistance to oxidative stress. As a result of these important discoveries, and thanks to the ease to work with mutants and to manipulate its genome, *C. elegans* was exploited to show that CR induced modulation of life-span is strictly correlated with mitochondrial integrity [31,61,62], revealing the central role of mitochondria in the determination of metabolic plasticity.

Interestingly, the availability of the wide array of experimental tools, together with the possibility to control the bacterial food and the presence of specific nutrients, allowed the dissection of the molecular mechanisms involved in the CR dependent life-span extension, showing that different dietary regimens can modulate life-span by independent or overlapping mechanisms [63]. As a result, it is now widely accepted that the effects of CR are caused by the interference with a network of genetic pathways rather than by with a single, linear pathway.

*C. elegans* was also employed in the first studies on the dissection of the molecular pathways affected by resveratrol [21], confirming AMPK as energy sensor responsible of life-span extension, and guiding the search and screening of new CR-mimetics [64].

Although the use of *C. elegans* in the study of longevity lead to important discoveries, this organism in not free of weaknesses. Being the body organization very simple, and due to the fact that the mechanisms involved in the beneficial action of CR can differ in the different organisms [31], its correlation with humans has to be cautiously evaluated.

## 4. *Drosophila melanogaster*

Used for the first time in aging experiments in 1916, demonstrating that its life-span was food and temperature dependent [65], *Drosophila melanogaster* is still considered a suitable model in aging research. Thanks to the feasibility of performing largescale screens for demographic analyses, the fruit fly has a bridging role in the validation of findings discovered in other model organisms [45,66]. As a consequence, *D. melanogaster* is largely exploited in the study of CR mechanisms and in the search of CR-mimetics [21,67].

At 25 °C, *D. melanogaster* has a life-span of approximately 60 days, which can be reduced increasing the temperature, and vice versa, increased by reducing the growth temperature. *D. melanogaster*, as other insects, can enter a reproductive diapause following light cycle and temperature modulation. As reported for *C. elegans*, diapause is connected to a better stress resistance and increased life-span.

*D. melanogaster* has been described to undergo functional senescence at tissue and organ level [68]. Interestingly, similarly to what has been observed in humans, during aging, flies present the alteration of selected biomarkers as the advanced glycation end products, or carbonylated proteins [69]. At the organ level, age progression induces unbalanced gut homeostasis, altered cardiac and skeletal muscle function, and neurological and neurosecretory modifications [70,71]. More importantly, although it is needless to stress that *D. melanogaster* is far from mirroring the human organism, it is an excellent model to study the genetic complexity of the aging process.

From the laboratory point of view, *D. melanogaster* requires a simple and cost-effective maintenance, and researchers can acquire a very good knowledge on the factors that should be considered when working with the fruit fly [68]. The simplicity of its genome, consisting of 13,000 genes belonging, approximately, to the same mammalian gene families [45], together with the availability of strains with the same genotype, facilitate demographic analyses and the performance of largescale screens [45,66]. Actually, *D. melanogaster* is an excellent model to perform genetic analyses thanks to the availability of mutant and transgenic strains, and the accessibility to temporal, hormone-inducible, and tissue-specific expression of mutated proteins [72]. Not last, a collection of RNAi lines enables the targeting of most transcripts to perform knockdown screens [73].

The above-mentioned characteristics make the fruit fly an experimental model often exploited to delve into the genetics of CR, and into the search and characterization of CR-mimetics [67]. Life-span prolongation upon CR in *D. melanogaster* has been shown to be mediated by five mechanisms: the cotransporter encoded by Indy [74], the insulin/GF-like signaling pathway [75], the Rpd3 deacetylase [76], the dSir2 deacetylase [77], and TOR signaling pathway [78]. The broad knowledge of the CR restriction mechanisms in *D. melanogaster*, together with the relative ease to work with this organism allowed to confirm resveratrol as a CR-mimetic, and to screen interventions with potential effects in life-span extension [79,80].

Although *D. melanogaster* is still far from the human organisms, this experimental model has supplemental advantages compared to yeast and *C. elegans* that justify its employment in the study of CR. It is an obligate aerobe, and it is dioecious, two factors that can have an important impact on aging [81], and on the response to anti-aging measures, making *D. melanogaster* a model organism suitable to perform both genetic manipulation and physiological analyses.

## 5. Fishes

The fishes include the shortest- and longest-lived vertebrates in nature [82,83,84]. The heterogeneity in longevity provides a unique possibility for exploring the molecular mechanisms that determine the differences in the rate of aging by applying comparative studies [85,86].

In the view of the laboratory application, the small tropical fish species are considered to have the best potential in aging research, since they display a short life-span, gradual senescence, and development of degenerative processes and tissue lesions in an age dependent way [87,88]. Although the zebrafish (*Danio rerio*) is the most utilized model system in the research laboratories, guppy and killifish have a potential in aging research due to their short life-span [87,89].

Widely employed in developmental studies, the zebrafish is increasingly used in aging research, due to its relative short life-span (2–3 years), and based on the evidence that it shows hallmarks of gradual senescence, such as spinal curvature, muscle degeneration, and reduced physical ability [90,91,92]. Interestingly, recent research shows that zebrafish can be exploited for the investigation of neurodegenerative pathologies as Alzheimer disease, Parkinson disease [93], but also osteoporosis, sarcopenia [94], and age-dependent trainability [95].

Zebrafish have several advantages for routine research because housing is quite simple, it has a good reproductive capacity, and can provide sufficient amounts of tissues for sampling. Zebrafish have a conserved genome, which is easily modifiable [96]. Moreover, research with zebrafish is greatly supported by the availability of well-established methodological and biological tools, spanning from genetic manipulation [96], live imaging [97], adaptation to high-throughput screenings [98,99,100]. Moreover, as discussed for *D. melanogaster*, zebrafish can be exploited for demographic studies.

For the above-mentioned reasons, zebrafish have an interesting potential as a model to investigate CR and CR-mimetics. Interestingly, zebrafish feeding conditions can be easily modified upon need, providing the possibility to control the diet to reduce the caloric intake [101,102], or to induce obesity [103], causing genetic modulations similar to those observed in mammals. Likewise, zebrafish is largely used to test CR-mimetics and in the investigation of resveratrol for its CR-mimetic properties [104,105].

The above-mentioned features together with the fact that this is a more complex organism with defined organs and apparatuses, confer to zebrafish a bridging role between more simple organisms and mammals, in the search for anti-aging molecules.

## 6. Rodents

Rodents are the most common mammalian used in research, and include species with different life-spans, such as mice, rats, naked rat, moles, and others. To date, the mouse and the rat models are the most exploited mammalian models used in aging research, and have enabled important progress in the field of CR and CR-mimetics. Actually, the CR capability to induce life-span elongation was demonstrated for the first time by McCay and collaborators on albino rats [4], and the potential of resveratrol as CR-mimetic was demonstrated in C57BL/6 mice. Despite the awareness of multiple differences with the human aging [28,29], the most used animal model are mice, and, here, we attempt to shortly describe the main advantages and disadvantages of the use of mouse models in the perspective of planning experiments to delve into CR and CR-mimetics potential in attenuating aging.

Mice have about 3 years life-span, with slight changes depending on the strain, with inbred strains being more prone to aging [106,107]. Mice are quite similar to humans in their physiology, cellular functions, and, to a lesser extent, in their anatomy. Mouse aging has been shown to cause changes in many organ systems, in the body composition, in the cognition, and to induce a decline of the physical function [106,107]. The genome of the mouse, with 2.5 Gbp and 40 chromosomes, encodes almost the same number of genes of the human genome, with the 99% of the mouse genes having a human orthologue. Mice are available as inbred strains, therefore they have a genomic homogeneity [45]. To date, there is a large availability of mutant strains and gene-modifying tools that allow to delve into multiple cellular mechanisms. Moreover, the rapid advances in gene editing techniques have made the development of mutant strains less complicated and more rapid compared to the past.

In the context of the aging and CR research, models as the Ames and the Snell dwarf mice have demonstrated to have a longer life-span, ascribable to naturally occurring point mutations to the Prop1 gene [108] and Pou1f1 [109] that, similarly to what demonstrated in yeast, *C. elegans* and *D. melanogaster*, envisage an alteration of the insulin/IGF-1 pathway [110,111]. On the other hand, the availability of strains that display obesity [112], or accelerated aging [113,114,115], provides a further tool to investigate, in a restricted time-window, the effects of CR regimens and CR-mimetics.

A further advantage in adopting mice as an experimental model in the study of CR is the tuneability of the feeding conditions that allow to simulate obesity or diabetes, but also different nutrition-dependent contexts [112,116]. In this context, C57BL/6 mice fed with a high fat regimen revealed the CR-mimetic properties of resveratrol in mammals, showing that this small polyphenol is able to mimic some molecular aspects of the CR regimens and to improve the health-span in mice by reducing IGF-1 levels, increasing insulin sensitivity, AMPK and PGC-1alpha activity, and mitochondrial activity [24,117]. At the physiological level resveratrol has been showed to induce protection against type II diabetes [23,24], cardiovascular diseases [118,119], and improve the skeletal muscle functions [25,120,121,122]. Further data on resveratrol have been obtained by exploiting different mouse models, such as naturally aging C57BL/6 mice, diabetic mice [123], senescence-accelerated mice [124], and transgenic strains [125].

This being said, the exploitation of mice in the investigation of CR and CR-mimetics on the whole organism could seem perfect. However, rodents and humans differ both for basic biological process (i.e., regulation of telomere length, the DNA repair mechanisms, and the immune response), and life conditions (i.e., diet composition, physical activity, and life in a restricted state), that, in turn, have a different impact on aging and on the effects of CR and CR-mimetics [126].

## 7. Nonhuman Primates

Although working with the above-described experimental models certainly has multiple advantages, the differences with human aging prevent the direct translation of findings from model organism to humans. This gap could be overcome by the use of nonhuman primates, which are considered a good translational model because they have similar genetic, physiological, and behavioral characteristics to humans. Nonhuman primates display about 92% of genetic homology with humans, they exhibit age-associated dysfunctions and diseases, and are outbred with a high inter-individual variability similarly to humans [127,128]. The use of nonhuman primates has provided an important contribution in the study of vaccines, in transplant technology, in the study of infectious diseases, aging [129,130], and have been exploited to investigate the effects of CR in longitudinal protocols [131,132].

Nonhuman primates are grouped in two main categories, the Old World and the New World monkeys [130]. The Old World monkeys, which originate from Asia and Africa, have a medium to large size, variable life-spans, and include rhesus monkey, as macaques, that are the most utilized in aging research [129,130]. The New World monkeys, which originate from South America, have a smaller size and a shorter life-span. The most used New World monkeys in research are the marmosets, which live in multigenerational family units, allowing a simple maintenance. The smaller the size (200–450 g), the shorter the life-span, together with observation that these monkey display most of the aging characteristics observed in humans, means that nonhuman primates could represent a good opportunity to reduce costs and time, providing this model with a huge potential in aging research [130,131].

Fifty years after the first work on CR in rodents [4], at the end of the 1980s, CR experiments were applied on rhesus monkeys by different research groups, with the aim to perform longitudinal studies [133,134]. Since then, CR in nonhuman primates has been shown to have anti-aging properties [135,136], decrease the weight, ameliorate glucoregulation [137], reduce inflammation and cardiovascular diseases risk [135], and, in general, to improve the health-span [136]. In line with the necessity to use CR mimetics in patients that cannot afford a calorie restricted regimen, more recently, nonhuman primates have been largely employed in the study of resveratrol. Resveratrol has been demonstrated to improve several physiological parameters in nonhuman primates fed on a regular or modified diet [138,139,140,141], providing good chances for the translation to humans.

Although the nonhuman primates represent the most proximate model to humans, their use in the translational study of CR needs careful evaluation. If, on one side, there is an increasing knowledge of the molecular pathways determining longevity, nowadays there is also the awareness that life-span and health-span are modulated by complementary variables, including the macronutrients in the diet, physical activity, gender, and genetic background [126], which can modify the response to CR regimens.

## 8. Studies in Humans

Aging research in humans is complicated by the long life-span, the large variability among individuals, and multiple factors, such as socio-economic and cultural conditions, that can affect the aging process.

Studies on human aging can exploit the cross-sectional or the longitudinal design, but neither of them are free of flaws, and can lead to different interpretations [142]. Cross-sectional designs, which analyze different age groups at the same time point, are influenced by the dramatic changes occurred in the last 100 years of social, nutritional, and work conditions. Longitudinal studies, which follow the same individuals along time, reveal a high degree of individual variability, which is likely to depend on the social, nutritional, and work conditions changes, but also on the variation of the physio-pathological status experienced throughout the life-span of the individual.

Nowadays, the search for the effects of long-term lifestyle interventions initiated in early adulthood and carried on throughout the entire life captures much attention, due to the evidence that in some tissues and organs, such as the skeletal muscle [28], the functional decline can begin in adulthood. This interest has prompted several observational studies to understand the correlation between nutrition and health-span, and the potential of CR regimens and CR mimetics in improving the health-span of aging people. An example has been provided by Okinawans who are the world’s longest-lived population. The prolongation of the life-span of this population has been attributed principally to CR and the presence of CR mimetics in the diet [5,6].

At date, there is also a large number of studies aimed at directly testing CR regimens and CR-mimetics, but there are still some shadows on their efficacy [143,144], because the time and the interval of the intervention, the variability among individuals, and other factors can compromise their effectiveness [145].

Interestingly, also the investigation on humans can exploit an experimental model of accelerated aging. This is the bed rest model, which became very popular after spaceflights as a tool to investigate the effects of microgravity. The bed rest model is based on the evidence that microgravity and long periods of immobilization cause an alteration of mechano-skeletal and vestibulo-neuromuscular stimuli that have detrimental effects on the normal physiology of several organs and apparatuses, such as skeletal muscle, bones, cardiovascular system, and to unbalance several biomarkers [146]. Although bed rest experiments are restricted due to their nature, in recent years they have allowed the collection of an enormous amount of data, which contributed to expanding the knowledge on physiological changes during loss of gravitation, and on the mechanisms of aging.

Interestingly, aiming at simulating the nutritional condition of astronauts, who consume fewer calories, bed rest protocols envisaged the application of CR regimens, that have been shown to modulate several physiological parameters [147,148]. Interestingly, although CR enhances protein catabolism in 2-week inactive individuals [149], it prevents the inflammatory state induced by the inactivity [150].

## 9. Conclusions

The increase in the world’s aging population (https://population.un.org/wpp/; reporting the World population prospect of 2019, accessed on March 2021) has motivated a large effort in the investigation of the mechanisms underlying aging, and in the search of possible countermeasures. The search of strategies to improve the health-span in humans face with the intrinsic complexity of investigating in humans, such as the presence of ethical issues, and the difficulties to perform both longitudinal and cross-sectional studies. Mainly for these reasons, researchers opt in favor of using experimental models. Hence, the researcher has to deal with the evidence that, on one hand there are conserved mechanisms that regulated life-span, on the other, there are important variations in the fine mechanisms underlying aging in different organisms, between related species, and even among distinct individuals, conferring to aging a multifactorial nature [151].

Consequently, the cautious choice of one experimental model compared to another (Table 1), is based on the questions the researcher wants to answer. In the investigation of CR and CR-mimetics, the experimental models used are multiple, generally starting with a simple organism, and subsequently translating to more complex organisms. This approach has been exploited both for the study of several signaling pathways [14,16], and in the search of anti-aging agents [20,22]. Special attention has to be dedicated to the target tissue or organ, because senescence can affect tissue and organs dependently of the experimental model considered. It is important to mention that, the set-up, the availability of dedicated facilities, and the know-how of the laboratory are important in guiding the selection of the experimental model.

It is worthwhile to mention that, nowadays the researcher can afford new methods of analysis and new knowledge to tackle aging mechanisms and anti-aging interventions. These include silico analysis and simulations [152], and system biology studies [153], which can be of fundamental help when planning a research project on aging, and searching for interventions able to ameliorate life-span and health-span [151,154,155,156].

## Figures and Tables

**Table 1:** Summary of the most relevant properties of the described model systems.

	Yeast	Worms	Flies	Fishes	Rodents	Non-Human Primates
Life span	Very short	Very short with little fluctuation	Very short	Variable	Variable	Long
Genome	Restricted number of genes, fully characterized genome with a low similarity with human	Restricted number of genes, fully characterized genome with a low similarity with human	Restricted number of genes, fully characterized genome with a medium similarity with human	High number of genes, with a medium -high similarity with human	High number of genes, with a medium -high similarity with human	Similar number of genes, with high similarity with human
Anatomo-physiology	Unicellular	Very simple organism with tissues similar to human, hermaphrodite	Simple organism, with tissues similar to human, aerobe	Organism with defined organs and apparatuses	Mammals, with high similarity except some tissues and organs	Similar physiological and behavioral characteristics
Experimental tools	Easy genetic interventions	Easy genetic interventions, Mutant strains, RNAi libraries, Control of feeding conditions	Easy genetic interventions, Mutant strains, transgenic tunable strains, RNAi libraries, Control of feeding conditions	Easy genetic interventions, Mutant strains, transgenic tunable strains, RNAi libraries, Control of feeding conditions	Inbred strains, Control of feeding and diet conditions	High inter-individual variability similar to humans, Life in multigenerational family units
Major applications in CR research	Studies on mechanisms of CR, Screening of CR-mimetics, High-throughput analyses	Studies on mechanisms of CR, Screening of CR-mimetics, High-throughput analyses	Studies of mechanisms of CR, Screening of CR-mimetics, High-throughput and largescale screens for demographic analyses	Studies of mechanisms of CR, Screening of CR-mimetics, High-throughput and largescale screens for demographic analyses	Application of CR and CR-mimetics treatments in normal and pathological conditions	Longitudinal studies on CR and CR-mimetics
Pitfalls	Unicellular organisms, Restricted gene homologs	Different life cycle compared to mammals, Very simple organs	Different life cycle compared to mammals, Simple organs,	Different life cycle and habitat compared to mammals, Relatively simple organs and apparatuses, Dedicated facility	Different anatomo-physiological and aging properties of some organs and tissues, Dedicated facility	Long-term studies, Ethical concerns, Dedicated facility

## Data Availability

The study did not report original data.
